# Patient characteristics and early experiences with anti-obesity medications: the CAMP study

**DOI:** 10.3389/fnut.2026.1931188

**Published:** 2026-09-08

**Authors:** Colleen Tewksbury, Emma Adelia Soliva, Krista Scheffey, Pui Lui Kwong, Karen Glanz

**Affiliations:** 1 Department of Biobehavioral Health Sciences, School of Nursing, University of Pennsylvania, Philadelphia, PA, United States; 2 Department of Biostatistics, Epidemiology, and Informatics, Perelman School of Medicine, University of Pennsylvania, Philadelphia, PA, United States

**Keywords:** anti-obesity medications, GLP-1 receptor agonists, medication access, obesity, patient-reported outcomes

## Abstract

**Introduction:**

The widespread adoption of glucagon-like peptide-1 receptor agonists (GLP1-RAs) and related anti-obesity medications has transformed obesity treatment. However, limited data are available regarding the characteristics of patients receiving prescriptions for these medications and patients’ real-world experiences with medication access, utilization, and supportive care.

**Methods:**

The CAMP (Characteristics of Anti-Obesity Medication Patients) study was conducted in a large academic health system. Phase I characterized demographic, clinical, and social determinants of health among adults prescribed semaglutide or tirzepatide for weight management between July 2023 and June 2024. Phase II was an online survey assessing prescription fulfillment, medication use, payment methods, discontinuation, counseling, and referrals to additional services. Descriptive statistics and bivariate analyses were used to summarize findings and examine differences between patients receiving a prescription for the lowest dose versus the higher doses.

**Results:**

A total of 21,610 adults prescribed semaglutide or tirzepatide were identified through electronic health records in Phase I, and 2,628 completed the Phase II patient survey. Patients in Phase I were predominantly female, White, and privately insured, with a mean age of 49 years and mean BMI of 36 kg/m^2^. Most prescriptions were written by primary care providers. Among survey respondents in Phase II, 90.1% reported successfully filling their initial prescription and 80.5% remained on treatment. Among the 512 respondents who reported discontinued treatment, the most common reasons were insurance coverage discontinuation (37.7%), high medication costs (20.3%), and side effects (27.1%). Although 88.0% of respondents reported receiving medication-related instructions, only 27.0% reported receiving a referral to an additional health service, most commonly nutrition counseling.

**Discussion:**

Patients prescribed contemporary anti-obesity medications were predominantly female, White, and privately insured. Financial barriers were the main reasons for treatment discontinuation, and potentially important referrals to nutrition and behavioral services were infrequent. These findings provide important real-world insights into the implementation of anti-obesity pharmacotherapy and identify leverage points for improving access, continued use, and multidisciplinary obesity treatment.

## Introduction

Obesity is a chronic, relapsing disease that affects more than 40% of adults in the United States and is associated with increased risk of type 2 diabetes, cardiovascular disease, certain cancers, reduced quality of life, and premature mortality (1, 2). Despite decades of clinical and public health efforts, obesity prevalence and severity have continued to rise, highlighting the limitations of existing treatment approaches and the need for effective long-term therapies (3). Clinical practice guidelines recommend a comprehensive treatment approach that includes nutrition, physical activity, behavioral interventions, pharmacotherapy, and metabolic and bariatric surgery when appropriate, though it is not known how often these treatment components are used in practice (4–6).

The approval of highly effective anti-obesity medications (AOMs), particularly glucagon-like peptide-1 receptor agonists (GLP-1RAs) and dual glucose-dependent insulinotropic polypeptide (GIP)/GLP-1 receptor agonists, has transformed the landscape of obesity treatment (7). Semaglutide and tirzepatide have demonstrated substantial weight loss in randomized clinical trials, with average reductions in body weight exceeding those historically achieved with earlier medications and with intensive behavioral counseling (8, 9). In addition to weight loss, these medications have been associated with improvements in cardiometabolic risk factors and obesity-related comorbidities (10, 11). As a result, demand for these therapies has increased rapidly among patients and healthcare teams (12). While clinical trials have established the efficacy and safety of these medications, less is known about their use in clinical practice. Patients receiving AOMs outside of clinical trials may encounter challenges that are not fully captured in efficacy studies, including difficulties obtaining prescriptions, insurance coverage restrictions, high out-of-pocket costs, medication shortages, and barriers to ongoing treatment (12). Furthermore, little is known about the extent to which AOM patients receive nutrition and behavioral counseling. Understanding patients’ real-world experiences is important for identifying barriers to treatment initiation and continuation, as well as opportunities to optimize clinical care.

Emerging studies have begun to examine real-world utilization patterns of GLP-1-based therapies; however, most have relied on administrative claims and have focused primarily on treatment continuation or clinical outcomes (12, 13). Comparatively few investigations have directly surveyed patients regarding their experiences obtaining and using these medications (14). Patient-reported information on medication access, affordability, side effects, discontinuation, and supportive care may provide valuable insights that complement clinical and administrative data (15).

To address these gaps, the CAMP (Characteristics of Anti-Obesity Medication Patients) study was developed as a three-phase investigation within a large academic health system. The study aims to characterize patients prescribed AOMs and examine their experiences with medication access, insurance coverage, side effects, weight-related outcomes, weight management history, and psychosocial factors related to treatment. The present article reports findings from the first two phases of the study. Phase I used electronic health record (EHR) data to characterize patients prescribed semaglutide or tirzepatide for obesity treatment, and Phase II surveyed patients regarding their experiences obtaining and using these medications in routine clinical practice. Together, these findings provide a valuable real-world perspective on the characteristics and early treatment experiences of patients receiving contemporary AOMs.

## Materials and methods

### Study design

The CAMP study was conducted within the University of Pennsylvania Health System, a large academic health system in Philadelphia, Pennsylvania. The study consisted of three sequential phases. Phase I used EHR data to characterize patients prescribed AOMs within a large academic health system. Phase II surveyed patients regarding their experiences obtaining and using prescribed AOMs. A secondary objective of Phase II was to identify participants who were eligible for and willing to participate in Phase III. Phase III is a more comprehensive survey examining affordability, access, and psychosocial experiences related to weight management and AOM use. This paper reports on the first two phases of this study.

### Phase I

For Phase I, data were extracted from the EHR. Adult patients (≥18 years) were included if they received a prescription for semaglutide (Wegovy or Ozempic) or tirzepatide (Zepbound or Mounjaro) for weight management between July 2023 and June 2024. Demographic and clinical characteristics were obtained from the EHR for descriptive analyses. The University of Pennsylvania Institutional Review Board approved a waiver of HIPAA authorization for this phase, and no identifiable protected health information was included in the analytic dataset.

### Phase II

Patients identified in Phase I were screened for eligibility for the Phase II survey. Eligible participants were adults aged 18 years or older who had received at least one AOM prescription during the study period and had indicated they were willing to be contacted for research in their EHR profile.

For Phase II, eligible patients were invited via email to participate in an online survey hosted in REDCap. The invitation included a brief description of the study and a unique survey link. Participants were not compensated for survey completion. A waiver of HIPAA authorization was also approved for inviting patients to participate in Phase II. Because the study was determined to be minimal risk, informed consent was obtained through an electronic consent process at the beginning of the survey. Participants who selected “Yes, I agree to take this survey” were granted access to the survey questions, whereas those selecting “No, I do not agree to take this survey” exited the survey and provided no additional data. Following pilot testing and refinement of the survey instrument, recruitment emails were distributed in batches of approximately 1,000 patients per week. Non-responders received up to three reminder emails. Data collection took place between March and August 2025.

### Variables and measures

For Phase I, demographic, clinical, and social risk variables were extracted from the EHR. Variables included age, sex, race/ethnicity, body mass index (BMI), and weight. Clinical characteristics included prescribing clinician department, pharmacy type (hospital vs. community), primary payer, smoking status, and documented comorbidities including hypertension, dyslipidemia, cardiovascular disease, and sleep apnea. Prescribed AOM type was also captured. Social determinants of health measures routinely documented in the medical record included financial resource strain, housing stability, food insecurity, and transportation needs. Additional behavioral and psychosocial indicators available in structured EHR fields were extracted, including physical activity level, stress, and depression risk. All variables were obtained as recorded in the medical record during the study period. For patients for whom there were multiple records in the dataset, the first encounter date was used for analysis.

For Phase II, patient-reported data were collected through a structured REDCap-based questionnaire assessing experiences with AOM prescribing, access, initiation, and use. Survey items included whether participants were able to fill their initial prescription for a weight loss medication and reasons for not obtaining the medication when applicable. Current medication use was assessed by asking whether participants were actively taking a weight loss medication at the time of survey completion, as well as reasons for discontinuation for those no longer taking an AOM. Participants identified the name of their initially prescribed AOM, reported whether they received their medication, and the payment source for the prescription when applicable. The survey also assessed receipt of medication-related counseling and supportive care, including whether patients received referrals to other health services, the types of referrals received, and whether these referrals were completed. Additionally, participants reported whether they received instructions from their prescribing clinician regarding medication use and dosing, with itemized response options capturing the specific types of instructions provided. For the purpose of data analysis and to keep the survey relatively short, demographic and clinical characteristics from the EHR (retrieved for Phase I) were linked to the survey responses, and included age, sex, race/ethnicity, and height and weight (to calculate BMI).

### Statistical analysis

For Phase I, EHR data were extracted and analyzed using SAS version 9.4 (SAS Institute Inc., Cary, NC, United States). After removing duplicate records for the same individuals, people were excluded if they received prescriptions from a non-Penn Medicine clinician, had implausibly low weight values in the medical record (<110 pounds), or had a recorded weight greater than 400 pounds. Descriptive statistics were used to characterize the study cohort overall and by medication type. Continuous variables were summarized using means and standard deviations, as appropriate based on the variable and data distribution. Categorical variables were summarized using frequencies and percentages. Exploratory analyses were conducted to describe patient demographics, anthropometric characteristics, prescribing clinician specialty, pharmacy type, insurance coverage, and social determinants of health measures available within the EHR. Cross-sectional analyses also compared patients on the lowest dose medications to those on higher doses at the time of the data extraction, using chi-square analyses and *t*-tests. Because the data pulled were a “snapshot” for a one-year period, this comparison compared patients who were prescribed only the lowest dose medications, potentially were earlier in their pharmacotherapy treatment or prescribed a lowest dose that was not filled, to those who received prescriptions for higher doses, indicating that they had been using AOMs for a longer time.

For Phase II, survey data were collected and managed using REDCap, a secure web-based application for research data capture. Survey responses were exported from REDCap and analyzed using SAS version 9.4. Descriptive statistics were calculated to characterize survey respondents and summarize responses related to medication access, prescription fulfillment, insurance coverage, affordability, medication use, discontinuation, side effects, and receipt of medication-related counseling and referrals. Continuous variables were summarized using means and standard deviations, and categorical variables were summarized using frequencies and percentages. Analyses were limited to available responses for each survey item; therefore, denominators may vary across survey questions due to some multiple-choice questions and item nonresponse.

## Results

### Phase I

There were 69,690 prescriptions identified with the initial parameters. Twenty-nine observations of patients younger than 18 years of age were excluded. If a patient had received multiple prescriptions captured in the data export, the first encounter date was used for analysis. As a result, 47,320 observations for patients with multiple prescriptions were removed. In addition, the following were removed: 555 outliers were removed due to weight higher than 400 lbs. (*n* = 223 patients) or lower than 110 lbs. (*n* = 332 patients), 251 with missing height and/or weight for calculated BMI, 54 with the same email addresses, and 172 with a duplicate medical record number.

The main analytic data set included 21,610 patients who received a prescription for semaglutide or tirzepatide for obesity treatment during the study period (Table 1). 12,512 (57.9%) received a prescription for the lowest medication dose, while 9,098 (42.1%) received higher dose prescription (Table 1). Patients in the lowest-dose prescription group had significantly higher mean BMI (36.9 ± 7.2 vs. 34.5 ± 7.7 kg/m^2^, *p* < 0.0001) and body weight (228.4 ± 51.1 vs. 213.1 ± 52.7 lbs., *p* < 0.0001), likely reflecting treatment stage. Age (48.6 vs. 48.8 years) was not significantly different between groups. Compared with patients prescribed higher doses, those prescribed only the lowest dose were less female (75.4% vs. 78.6%) and a greater proportion identified as White (65.6% vs. 62.1%) (both *p* < 0.0001). Prescriptions for the lowest dose were more frequently written by primary care providers (71.0% vs. 47.9%), whereas patients receiving higher dose prescriptions were more likely to come from endocrinology, bariatric/weight management, or other subspecialty providers (*p* < 0.0001). Patients in the higher-dose prescription group were also more likely to use hospital or mail-order pharmacies and to have Medicaid or other insurance coverage, while Medicare coverage was more common among those prescribed the lowest dose (*p* < 0.0001). Finally, medication type differed significantly between groups, with tirzepatide accounting for a larger proportion of prescriptions among patients prescribed the lowest dose (44.6% vs. 19.4%), whereas semaglutide was more common among patients receiving higher dose prescriptions (80.6% vs. 55.4%) (*p* < 0.0001). This was expected because tirzepatide was not approved by the FDA for weight management until November 2023, the 5th month of the one-year data pull.

**Table 1:** Characteristics of patients prescribed semaglutide or tirzepatide for obesity treatment (*N* = 21,610).

Variable	Lowest dose *N* = 12,512	Higher doses *N* = 9,098	*p*-value
Age, year, mean (SD)	48.6 (13.2)	48.8 (12.2)	0.4396
BMI, Kg/m^2^, mean (SD)	36.9 (7.2)	34.5 (7.7)	<0.0001
Weight, lbs., mean (SD)	228.4 (51.1)	213.1 (52.7)	<0.0001
Sex, *n* (col %)			<0.0001
Female	9,431 (75.4)	7,153 (78.6)	
Male	3,081 (24.6)	1945 (21.4)	
Race, white/nonwhite, *n* (col %), missing = 1,107			<0.0001
Non-white	4,076 (34.4)	3,275 (37.9)	
White	7,787 (65.6)	5,365 (62.1)	
Residential location, *n* (col %), missing = 345			<0.0001
Philadelphia county, PA	3,326 (27.0)	2,722 (30.4)	
Other counties, PA	5,278 (42.8)	3,407 (38.1)	
Other states	3,714 (30.2)	2,818 (31.5)	
Prescription written department, *n* (col %)			<0.0001
Primary care	8,889 (71.0)	4,354 (47.9)	
Endocrinology, diabetes, bariatrics, weight management	1900 (15.2)	2054 (22.6)	
All other subspecialties	1723 (13.8)	2,690 (29.6)	
Pharmacy, *n* (col %)			<0.0001
Community pharmacies	5,673 (52.8)	2,495 (42.5)	
Hospital pharmacies	4,624 (43.0)	3,024 (51.5)	
Mail order/home delivery pharmacies	444 (4.1)	348 (5.9)	
Insurance, *n* (col %), missing = 178			<0.0001
Private	10,229 (82.3)	7,525 (83.6)	
Medicare	1,377 (11.1)	759 (8.4)	
Medicaid and other	822 (6.6)	720 (8.0)	
Medication, *n* (col %)			<0.0001
Tirzepatide	5,584 (44.6)	1768 (19.4)	
Semaglutide	6,928 (55.4)	7,330 (80.6)	

Among patients with available social determinants of health (SDOH) and health behavior data, most reported low levels of social and economic risk (Table 2). Overall, there was large missingness within SDOH measures in the EHR. Measures of financial resource strain, housing stability, and food insecurity were similar between patients for only the lowest dose prescription and those receiving higher dose prescriptions, with no statistically significant differences observed. Approximately 85% of patients in both groups were classified as low risk for financial resource strain, nearly 89% were considered low risk for housing instability, and approximately 90% reported no food insecurity. Patients receiving higher dose prescriptions were slightly more likely to report unmet transportation needs than those with a prescription for only the lowest dose (4.3% vs. 3.2%, *p* = 0.0017). Smoking status also differed between groups, with a greater proportion of current smokers among patients prescribed the lowest dose (6.3% vs. 5.1%, *p* = 0.0005). Although statistically significant, differences in physical activity and stress were modest. Patients receiving higher dose prescriptions were slightly more likely to be sufficiently physically active (33.2% vs. 31.5%) and less likely to report any stress concerns (43.5% vs. 45.5%) compared with those prescribed the lowest dose (*p* = 0.038 and *p* = 0.029, respectively). Depression risk did not differ significantly between groups, with approximately 4–5% of all patients screening at risk for depression.

**Table 2:** Social determinants of health and health behaviors among patients prescribed semaglutide or tirzepatide for obesity treatment (*N* = 21,610).

Variable	Lowest dose *N* = 12,512	Higher doses *N* = 9,098	*p*-value
Financial resource strain, *n* (col %), missing = 8,153			0.4252
Low risk	6,606 (85.1)	3,962 (85.1)	
Medium risk	857 (11.0)	533 (11.5)	
High risk	298 (3.8)	160 (3.4)	
Housing stability, *n* (col %), missing = 8,153			0.6984
Low risk	6,654 (88.8)	4,040 (88.6)	
High risk	837 (11.2)	520 (11.4)	
Food insecurity, *n* (col %), missing = 8,153			0.6827
No food insecurity	7,140 (90.3)	4,312 (90.1)	
Food insecurity present	767 (9.7)	475 (9.9)	
Transportation needs, *n* (col %), missing = 8,153			0.0017
No transportation needs	7,843 (96.8)	4,717 (95.7)	
Unmet transportation needs	261 (3.2)	211 (4.3)	
Smoking, *n* (col %), missing = 26			0.0005
Never smoked	8,464 (67.7)	6,296 (69.3)	
Former smoked	3,249 (26.0)	2,324 (25.6)	
Current smoked	787 (6.3)	464 (5.1)	
Physical activity, *n* (col %), missing = 9,147			0.0381
Inactive	1,402 (18.1)	783 (16.6)	
Insufficiently active	3,901 (50.4)	2,372 (50.2)	
Sufficiently active	2,438 (31.5)	1,567 (33.2)	
Stress, *n* (col %), missing = 9,058			0.0290
No stress concern present	4,289 (54.5)	2,641 (56.5)	
Stress concern present	3,586 (45.5)	2036 (43.5)	
Depression, *n* (col %), missing = 1904			0.3038
Not at risk	11,157 (95.4)	7,664 (95.7)	
At risk	540 (4.6)	345 (4.3)	

Obesity-related comorbidities were common among patients prescribed semaglutide or tirzepatide (Table 3). Patients in the lowest dose prescription group had a higher prevalence of all assessed comorbid conditions, including type 2 diabetes or pre-diabetes (20.5% vs. 15.8%), hypertension (19.7% vs. 11.8%), dyslipidemia (18.2% vs. 10.8%), obstructive sleep apnea (5.1% vs. 3.6%), and cardiovascular disease (2.8% vs. 2.2%) (all *p* < 0.05), likely a reflection of treatment stage. Type 2 diabetes/pre-diabetes, hypertension, and dyslipidemia were the most common obesity-related comorbidities in both groups. Overall, these findings indicate that a substantial proportion of patients being prescribed AOMs had obesity-related cardiometabolic conditions, although the majority did not have documented diabetes or cardiovascular disease.

**Table 3:** Obesity-related comorbidities among patients prescribed semaglutide or tirzepatide for obesity treatment.

Variable	Lowest dose *N* = 12,512	Higher doses *N* = 9,098	*p*-value
Type II diabetes, *n* (col %)	2,562 (20.5)	1,434 (15.8)	<0.0001
Hypertension, *n* (col %)	2,471 (19.7)	1,078 (11.8)	<0.0001
Dyslipidemia, *n* (col %)	2,274 (18.2)	979 (10.8)	<0.0001
Obstructive sleep apnea, *n* (col %)	632 (5.1)	327 (3.6)	<0.0001
Cardiovascular disease, *n* (col %)	345 (2.8)	203 (2.2)	0.0152

### Phase II

In total, 1,234 individuals from Phase I had notes in their medical record that they preferred not to be contacted for research and 634 were excluded for missing or duplicate email addresses or if they had an outside prescribing clinician. Of the 19,742 patients invited to participate in the survey, 447 (2.3%) were excluded because the email was undeliverable (*n* = 90) or the individual declined participation. A total of 2,872 patients responded, yielding a response rate of 14.9%. Among respondents, 2,628 were eligible for analysis, while 244 were excluded after the first survey question because they had not received an AOM prescription since July 2023 and thus would not be able to answer questions about prescription filling and medication use.

A total of 2,628 patients completed the full Phase II survey (Table 4). Respondents had a mean age of 48.5 ± 12.0 years and a mean BMI of 35.0 ± 7.3 kg/m^2^. The sample was predominantly female (74.1%), non-Hispanic (96.3%), and White (74.4%). Semaglutide was the most commonly reported initial prescription (58.5%). Of all the respondents, 90.1% reported successfully filling their initial prescription; there was no significant difference between White and Black patients (90.1% vs. 90.7%). Insurance was the most frequently reported payment source (81.0%), although many patients also reported paying a copay (44.2%). Additional payment methods included manufacturer promotional coupons (21.6%), out-of-pocket self-payment (14.1%), and payments to compounding pharmacies (0.8%). At the time of survey completion, 80.5% of respondents reported that they were still taking semaglutide or tirzepatide (81.9% of White patients vs. 75.6% of Black patients; *p* = 0.01).

**Table 4:** Characteristics and prescription status of Phase II survey participants.

Variable	*N* (%), M (SD)
	*N* = 2,628
Age, years	48.5 (12.0)
Body mass index, kg/m^2^	35.0 (7.3)
Sex, female	2094 (74.1)
Race, missing = 92	
White	1887 (74.4)
Black	464 (18.3)
Other^a^	185 (7.3)
Ethnicity, missing = 35	
Non-hispanic	2,496 (96.3)
Hispanic	97 (3.7)
Initial prescription	
Semaglutide	1,537 (58.5)
Tirzepatide	961 (36.6)
Other^b^	130 (4.9)
Able to fill prescription	
Yes	2,369 (90.1)
No	259 (9.9)
If you received your prescription, how was it paid for? (multiple answers allowed)^c^	*N* = 2,369
Insurance	1918 (81.0)
Copay	1,046 (44.2)
Promotional coupon from drug manufacturer	511 (21.6)
Out of pocket (self-pay)	335 (14.1)
Paid to a compounding pharmacy	20 (0.8)
Other^c^	19 (0.8)
Currently taking semaglutide or tirzepatide	
Yes	2,116 (80.5)
No	512 (19.5)
Why are you no longer taking medication? (multiple answers allowed)	*N* = 512
Insurance stopped covering cost of medication	193 (37.7)
Side effects	139 (27.1)
Cost or copay was too high	104 (20.3)
I decided to stop using the medication	100 (19.5)
Other^d^	67 (13.1)
Out of stock at pharmacy	43 (8.4)
Medication wasn’t effective	32 (6.3)
I did not pick up the prescription	24 (4.7)

a Other races include Asian, American Indian or Alaska Native, Hispanic, Native Hawaiian or Other Pacific Islander, and Multi-racial.

b Other prescriptions include Saxenda, Contrave, Victoza, Qsymia, Phentermine, Rybelsus, Trulicity, and Topiramate.

c Other methods of payment include: Participating in clinical trials and samples from a doctor.

d Other reasons for not taking medication include reaching goal weight, health complications, and pregnancy or trying to conceive.

Among the 512 respondents who reported no longer taking their medication, the most commonly reported reason for discontinuation was loss of insurance coverage for the medications (37.7%, Table 4), and about one in five respondents cited high medication costs or copays (20.3%). Side effects were reported as a reason for discontinuation by 27.1% of respondents, and other often-cited reasons included a decision to stop using the medication (19.5%) or other reasons (13.1%). Less frequently reported reasons included medication shortages (8.4%), lack of effectiveness (6.3%), and failure to pick up the initial prescription (4.7%). These findings suggest that financial and insurance-related barriers were the most common challenges to continued AOM use in this population. There were no significant differences in reasons for discontinuation between White and Black respondents.

Only 27.0% of respondents (*n* = 709) reported receiving a referral to another health service at the time of prescription. Referrals were most commonly made to a dietitian or nutritionist (90.0%), followed by a behavior modification counselor or psychologist (12.7%), pharmacist (9.4%), support group (5.1%), or other services (5.5%). Among those patients who received a referral, 70.5% reported attending or completing the recommended service. Most survey respondents reported receiving medication-related education from their prescribing clinician, although referrals to additional supportive services were less common (Table 5). Overall, 88.0% of respondents indicated that they received instructions from their clinician when prescribed semaglutide or tirzepatide. Among those who received counseling, the most common topics included the medication dosing schedule (90.4%), how to administer the medication (83.7%), and potential side effects (83.5%). These findings suggest that while medication-specific education was routinely provided, referral to nutrition, behavioral, and other supportive services into AOM care was considerably less common.

**Table 5:** Referrals and instructions received by Phase II survey participants.

Variable	*N* (%)
	*N* = 2,628
Did you receive a referral to another health service when you received this prescription?	
No	1919 (73.0)
Yes	709 (27.0)
Did you attend/complete the referral?	
No	209 (29.5)
Yes	500 (70.5)
What referral did you receive? (multiple answers allowed)	
Dietitian, nutritionist	638 (90.0)
Behavior modification counselor/psychologist	90 (12.7)
Pharmacist	67 (9.4)
Support group	36 (5.1)
Other^a^	39 (5.5)
Did you receive instructions from your medical provider with your prescription?	
No	314 (12.0)
Yes	2,314 (88.0)
What instructions did you receive? (multiple answers allowed)	
Medication dose schedule	2092 (90.4)
How to administer medication	1937 (83.7)
Information on side effects	1933 (83.5)
Other^b^	70 (3.0)

a Other types of referrals include specialist doctors, weight loss clinics, and prevention programs.

b Other types of instructions include diet and nutrition, physical activity, follow-up care, and expectations for long-term use.

## Discussion

In this large, two-phase cross-sectional study of patients prescribed semaglutide or tirzepatide within a major academic health system, we characterized both the demographic and clinical profile of patients receiving AOM prescriptions and their early experiences obtaining and using these therapies. Several important findings emerged that should be interpreted as cross-sectional observations of real-world prescribing practices and experiences, not treatment effects. First, patients receiving AOM prescriptions were predominantly female, two-thirds White, and most had private health insurance. Second, the majority of prescriptions originated from primary care clinicians rather than obesity medicine specialists. Third, although most patients reported receiving medication-related education, few were referred to additional supportive services such as nutrition or behavioral counseling. Finally, despite receiving pharmacologic treatment for obesity, some patients reported behavioral, psychosocial, and social risk factors that might influence treatment use and/or outcomes, including insufficient physical activity, stress, financial strain, food insecurity, and housing instability. It is noteworthy that, although social determinants of health questions are standard in the health system’s EHR, these data were missing for nearly 40% of patients—with the exception of questions about tobacco use and depression.

The demographic profile of patients receiving AOM prescriptions raises questions regarding access and equity. Although obesity disproportionately affects racial and ethnic minority populations and individuals with lower socioeconomic status, the majority of patients receiving prescriptions in this study were White and privately insured (16). However, we cannot determine whether the one-third of non-White patients reported as receiving AOM prescriptions in Phase I is representative of the race/ethnicity of the health system adult patient population with obesity, so interpretation of this finding must be done with caution.

More than 80% of patients receiving an AOM prescription had private insurance coverage, while relatively few were covered by Medicare or Medicaid. Medicare patients were not eligible for AOM coverage at the time of the Phase I data pull. As with race/ethnicity, we cannot determine how many patients with obesity and on Medicaid sought care in this health system. Still, these findings are consistent with previous reports including from Washington et al. that the population currently receiving GLP-1-based AOM may not fully reflect the broader population that is clinically eligible to benefit from these therapies (16). Insurance coverage restrictions, prior authorization requirements, and out-of-pocket costs may contribute to disparities in access, particularly among populations already experiencing disproportionate obesity burden (17). As AOMs become more widely adopted, continued monitoring of equitable access will be critical to ensure that advances in obesity treatment do not widen existing health disparities.

A notable finding was that more than 70% of the lowest dose prescriptions were written by primary care clinicians, highlighting the increasingly central role of primary care in obesity management. Historically, obesity pharmacotherapy was most often prescribed by specialists in endocrinology, obesity medicine, or bariatric programs (4). The rapid growth in demand for GLP-1-based therapies appears to have shifted much of obesity treatment into primary care settings (18). This trend underscores the need for accessible clinical resources, clinician education, and interdisciplinary support systems that enable primary care clinicians to offer or refer patients to supportive obesity care, including nutrition assessment as highlighted in the survey findings.

The behavioral and psychosocial characteristics observed in this cohort further demonstrate the complexity of obesity treatment beyond medication prescribing. Despite standardized, validated screening measures being included in the EHR, more than one third of participants were missing these data. This is a data gap and also highlights the need for more clinician adherence to social needs screening. Due to this, statistical significance should be interpreted with caution against clinical relevance. Only approximately one-third of patients with available physical activity data were classified as sufficiently active, while half were insufficiently active and nearly one in five were inactive. Although most patients reported low risk for adverse social determinants of health, a minority of those with data in their records reported experiencing financial strain, food insecurity, housing instability, or transportation barriers. While these findings are limited by inconsistent screening and recall bias, these challenges may influence patients’ ability to access medications, adhere to treatment recommendations, and engage in healthy lifestyle behaviors (17, 19). Similarly, nearly half of patients with responses in their records reported concerns related to stress and approximately one in twenty screened at risk for depression. While interpreting these data with caution given missingness and the cross-sectional nature, these findings reinforce the importance of viewing obesity as a multifactorial chronic disease influenced by biological, behavioral, psychological, and social factors.

The survey findings revealed a notably low rate of referral to supportive services along with medication prescribing, most strikingly the relatively low reported nutrition referral rate. While nearly 90% of respondents reported receiving instructions regarding medication administration, dosing schedules, and side effects, only 27% reported receiving a referral to another health service. Among those referred, nutrition services were by far the most common referral destination. Current obesity treatment recommendations emphasize that AOMs should be used as part of a comprehensive treatment strategy that includes nutrition, physical activity, and behavioral interventions although experts continue to debate what is most helpful due to gaps in knowledge (20–23). This finding highlights a significant gap in real-world practices when compared to current nutrition referral recommendations. There are clearly opportunities to better integrate multidisciplinary care into obesity treatment. Further studies should assess the reasons for low nutrition referrals, including potential access issues, lack of insurance coverage, gaps in knowledge, or possibly clinical assessment of lower nutritional risk by the prescribing provider.

Several limitations should be considered when interpreting these findings. Phase I relied on data available within the EHR and was therefore limited by the completeness and accuracy of routinely collected clinical documentation. The study design, in which we took a one-year “snapshot” of AOM prescriptions, left gaps in information about patients who had begun weight management medications before the start date; but also provided an opportunity to compare characteristics of people prescribed only the lowest dose vs. higher doses (Tables 1–3). There could be many factors influencing why a patient may only have a prescription for the lowest dose only that would not be captured in the EHR for this first phase, including ability to fill the prescription, discontinuation, or timing of the prescription in relation to the data collection. For the entire Phase I sample, key variables such as access, medication adherence, out-of-pocket costs, weight loss outcomes, and patient perceptions were not available through the EHR. In addition, several social determinants of health variables had substantial missing data. Phase II relied on self-reported survey responses of a subset of patients who responded and are subject to response bias. While the sample was statistically reflective of the Phase I participants, the low response rate should be interpreted with care and may limit the generalizability of these findings. Survey respondents were more female, White and had a slightly lower BMI when compared to eligible non-responders; this may have influenced the findings for Phase II bias. Furthermore, the study was conducted within a single United States-based academic health system, which may limit generalizability to other healthcare settings and patient populations.

The next phase of the CAMP study will extend these findings by examining patients’ weight management histories and longer-term patient experiences with AOMs, including treatment satisfaction, medication persistence, weight stigma, and social interactions related to AOMs. As the use of GLP-1-based AOMs continues to expand, real-world data from patients will be essential for informing clinical practice, reducing barriers to care, and optimizing outcomes for populations living with obesity.

## Data Availability

The raw data supporting the conclusions of this article will be made available by the authors, without undue reservation.
